# Fluorescence Microscopy—An Outline of Hardware, Biological Handling, and Fluorophore Considerations

**DOI:** 10.3390/cells11010035

**Published:** 2021-12-23

**Authors:** Shane M. Hickey, Ben Ung, Christie Bader, Robert Brooks, Joanna Lazniewska, Ian R. D. Johnson, Alexandra Sorvina, Jessica Logan, Carmela Martini, Courtney R. Moore, Litsa Karageorgos, Martin J. Sweetman, Douglas A. Brooks

**Affiliations:** Clinical and Health Sciences, University of South Australia, Adelaide 5000, Australia; christie.bader@unisa.edu.au (C.B.); robert.brooks@unisa.edu.au (R.B.); joanna.lazniewska@unisa.edu.au (J.L.); ian.johnson@unisa.edu.au (I.R.D.J.); alexandra.sorvina@unisa.edu.au (A.S.); jessica.logan@unisa.edu.au (J.L.); carmela.martino@unisa.edu.au (C.M.); courtney.moore@unisa.edu.au (C.R.M.); litsa.karageorgos@unisa.edu.au (L.K.); martin.sweetman@unisa.edu.au (M.J.S.); doug.brooks@unisa.edu.au (D.A.B.)

**Keywords:** fluorescence microscopy, microscopy techniques, imaging agents, cellular imaging

## Abstract

Fluorescence microscopy has become a critical tool for researchers to understand biological processes at the cellular level. Micrographs from fixed and live-cell imaging procedures feature in a plethora of scientific articles for the field of cell biology, but the complexities of fluorescence microscopy as an imaging tool can sometimes be overlooked or misunderstood. This review seeks to cover the three fundamental considerations when designing fluorescence microscopy experiments: (1) hardware availability; (2) amenability of biological models to fluorescence microscopy; and (3) suitability of imaging agents for intended applications. This review will help equip the reader to make judicious decisions when designing fluorescence microscopy experiments that deliver high-resolution and informative images for cell biology.

## 1. Introduction

Since the inception of fluorescence microscopes in the early 1900s, their use as a research tool for observing discrete subcellular structures and processes has grown immensely [1]. Initially, auto-fluorescent specimens were visualised with fledgling fluorescence microscopes, until the introduction of fluorescent stains in the 1930s, which enabled non-fluorescent specimens to be visualised [2]. The use of fluorescently-labelled antibodies in the 1940s [3] enabled precision visualisation of target structures in cells, and with the Nobel prize winning discovery of green fluorescent proteins (GFP) in the 1960s and their subsequent development as a genetic tag [4], the field has evolved to permit targeted live cell imaging. The discovery of novel fluorescent markers has driven the development of fluorescence-competent microscope technologies, further enabling researchers to discover and understand the intricate dynamics of subcellular biology and their detailed mechanics at ever-higher spatial and temporal resolutions (Figure S1).

**Figure 1:**
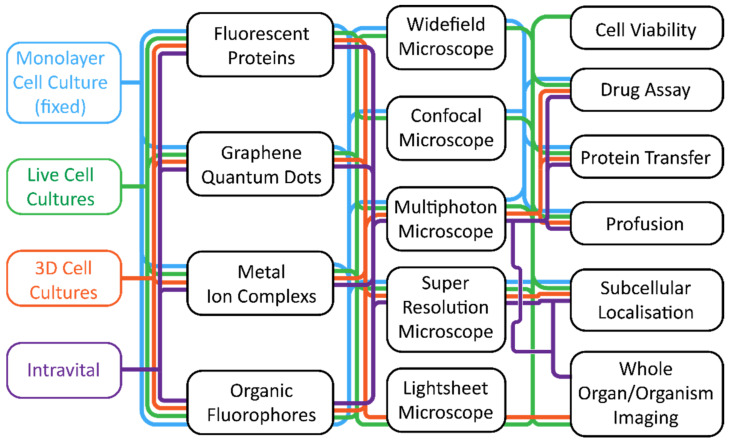
A flowchart linking biological systems to hardware combinations and applications. This flowchart should be read either from left to the right, working from the biological system to the desired application, or from right to left, where the application is chosen first. The flowchart is colour coordinated, and a chosen biological system will use the same colour throughout (blue for monolayer cell cultures, green for live cell cultures, orange for 3D cell cultures, and purple for intravital systems).

The rapid advances in the field of fluorescence microscopy have required a collaborative effort; from physicists and engineers developing microscopy hardware, to chemists that continually develop and refine novel fluorescent probes, and biologists who utilise these tools to investigate the biological functions of diverse specimens. A misunderstanding of some of these disciplines by researchers from other areas often leads to poor experimental design and research outcomes. This review aims to summarise the three main considerations when fluorescence microscopy experiments are employed: (1) current hardware availability; (2) the different biological models applicable for fluorescence microscopy; and (3) the plethora of fluorescent probes available. The advantages and limitations for each factor will be discussed to allow the reader to make informed decisions when contemplating experimental design. A helpful flowchart (Figure 1) is also provided to facilitate researchers to select the most appropriate combination of hardware, biological system, and imaging agent to achieve the best possible outcome in their imaging experiments.

## 2. Fluorescence Microscope Hardware Systems

Widefield epifluorescence microscopes, established during the early 20th century, represent the fundamental type of fluorescence technology [1]. Many incremental improvements have been made over the years, both to the efficiency of the optics, as well as excitation sources and detection systems/cameras. These fluorescence microscopes are still widely used and are amenable to a wide range of applications, including evaluating expression rates of fluorescent tags and visualising whole tissue mounts.

Confocal microscopy represents a next step in fluorescence microscopy, offering higher spatial resolution, with the ability to visualise subcellular details, which are unachievable by conventional widefield systems. Invented by Minsky in 1955 [5,6], the confocal microscope makes use of lasers to raster scan an image to a point detector, and with a pinhole, blocks unfocussed light from the imaging plane, enabling optical sections of samples to be imaged [7]. This forms the basis of other models of laser scanning microscopes, which are tailored to specific applications.

### 2.1. Multiphoton and Other Advanced Microscopy Techniques

Modifying the laser excitation source of a confocal microscope with an ultra-fast pulsed laser enables two- or three-photon microscopy [8]. Ultra-fast lasers allow deeper penetration of samples using longer excitation wavelengths, and optical sections are created through confinement of the two (or multi) photon effect to a single focal plane. This technique is suited to imaging large tissue samples, where penetration depths of 100 μm or more are needed to investigate macro-scale biological processes. Multiphoton microscopes are also needed for intravital imaging, where both gentle illumination and imaging depth are critical for imaging live whole organisms. However, multiphoton microscopes are limited by the added expense of purchasing an additional ultra-fast laser, which can add significantly to the cost of ownership and operation.

Laser scanning microscopes have been adapted to observe dynamic events in cellular function and exploit the properties of imaging probes, such as fluorescence lifetimes, and their interactions within and between cells. The three most common technologies employed are fluorescence lifetime imaging microscopy (FLIM) [9], fluorescence resonance energy transfer (FRET) [10], and fluorescence recovery after photobleaching (FRAP) [9]. FLIM requires the observation of intensity changes in a fluorophore over a course of time, which has been useful for quantifying decay rates of cell metabolites [11]. FRET measures energy transfer from one fluorophore to another using a single excitation, which allows for a variety of applications, such as calcium imaging [12] and protein–protein interactions/transfers [13]. Finally, FRAP measures the recovery in signal of a molecular probe after photobleaching, which enables the observation of diffusion kinetics in both cells and tissue samples [14].

Another recent technique, used to image larger tissue samples quickly and efficiently, is light-sheet fluorescence microscopy (LSFM) [15]. Light-sheet microscope operation differs from confocal microscopy, as samples are excited using a sheet of light from the side, as opposed to a focussed beam of light from the top or bottom of a sample. Emitted light is then detected perpendicular to the excitation sheet, unlike a confocal microscope, which detects the emitted signal in the opposite direction to the excitation beam. The thickness of the sheet of light determines the thickness of the optical section captured, and the sample can be rotated and moved through this plane to produce a three-dimensional tomographic image. Light-sheet fluorescence microscopy enables imaging of larger, multicellular samples, such as organoids and whole organisms (e.g., insects, plants, and animals [16,17,18]) that may not be adequately captured in a timely manner using single or multi-photon microscopy, although this does require tissue-clearing methodology to create optically transparent samples [19,20,21].

### 2.2. Super Resolution Microscopy (SRM); Going beyond the Limits of Light

Improvements in laser scanning microscopes have enhanced imaging resolutions beyond Abbe’s limit (Equation (1)), the physical limitation due to the physics of diffraction, for example, a 200 nm resolution limit in air when using an excitation wavelength of 400 nm [22]; however, compromises such as hardware cost, phototoxicity, and low capture speed, remain limitations. The simplest technique to improve resolution using a confocal microscope is to restrict pinhole size, perform a z-stack, which involves combining multiple images captured at sequential focal planes, and perform post-processing of images using software deconvolution. However, acquiring a suitable z-stack of images involves repeated laser excitation, which significantly increases both cellular phototoxicity and the time to capture the complete micrograph [23].

(1) $$d=\frac{\lambda }{2n\sin\theta }=\frac{\lambda }{2NA}$$

Equation (1) is Abbe’s formula for the resolution limit, where *d* is the minimum distance that can be differentiated between two points, *λ* is the wavelength of light, *n* is the refractive index of the medium the light is traversing, *θ* is the angle at which the light is converging, and *NA* is the numerical aperture [22].

Another approach to improve resolution is stimulated emission depletion (STED) [24], which makes use of two lasers; one for excitation, and the other shaped into a ring or doughnut, used as a de-excitation or depletion spot to limit the size of the emitted fluorescent spot. STED typically improves resolution to 100 nm in all three axes, but this has compromises with phototoxicity and a lack of compatibility with conventional fluorophores and involves an increase in system cost.

A unique hardware addition by ZEISS to improve image resolution involves the use of an AiryScan detector in their confocal microscopes [25]. The AiryScan uses an array of detectors laid out in a honeycomb pattern, which retains the light collection sensitivity of a conventional confocal microscope but enables an increase in resolution of approximately 1.4-fold without modifications to sample preparation. The compromise for this enhanced imaging technique is currently the increased hardware cost, and acquisition and processing time; the latter of which improves with continuous computational advances [25,26].

Additionally, structured illumination microscopy (SIM) is a hardware-based solution to improve image resolution by up to two-fold [27]. SIM works by projecting a moving grid onto the sample and recording multiple images as this lattice moves, creating an interference pattern known as the Moiré effect [27]. Processing these images improves the resolution by a factor of two, and recently, further improvements to the image processing algorithm have enhanced this to an additional two-fold [28], providing a spatial resolution as low as 60 nm. An advantage of SIM technology is the use of digital cameras for detection, rather than single point detectors, enabling these microscopes to capture highly dynamic events at frame rates exceeding 100+ frames per second [29]. Although SIM has minimal phototoxicity effects, it is limited by light penetration to maximal depths of ~200 μm [30,31].

Turn-key SIM systems can have the capability of performing single molecule imaging using photo activated localisation microscopy (PALM) or stochastic optical reconstruction microscopy (STORM). PALM and STORM both rely on photoactivation of fluorophores, which is a complex and highly sample-dependent process. Utilising this photoactivation of the fluorophores, molecules can be made to “blink” at differing time points and localities. Multiple images can then be acquired, and together with specialised algorithms, these images can be processed to achieve resolutions as low as 20 nm [32]. However, the current hardware cost of SIM systems limits their widespread adoption.

Microscope hardware is ever-evolving to suit the needs and applications of cell biologists. With steadily improving flexibility, sensitivity, resolutions, and speed, many historical challenges have been overcome, opening opportunities for further exploration and discovery. The key challenges are to minimise the trade-offs associated in choosing specific solutions, particularly with speed, resolution, phototoxicity, and imaging depth. To help the reader better understand the options available, we have compiled a detailed and useful table which compares the microscope types with respect to their advantages and limitations (Table S1).

## 3. Biological Models for Fluorescence Imaging; from the Monolayer Culture to the Whole Organ

The improvement in microscope technology allows biologists to employ a range of suitable models for the investigation of cellular mechanics. Cell culture plays a pivotal role in many fluorescence microscopy applications, where cells can be imaged from monolayers to three-dimensional (3D) cultures, such as spheroids, organoids, and tissue section explants, or extended to in vivo intravital imaging on living animals. In this section, we discuss the requirements and caveats of cell/tissue culturing to optimise the potential for imaging.

### 3.1. Monolayer Cell Culture

There are over 4000 well-characterised, readily available cell lines serving as models for human disease and development, which can be studied by researchers using a variety of imaging applications. Cell culture systems provide several advantages as models of more complex biological systems, including their potential for high-throughput screening, reproducibility, cost-effectiveness, reduced ethical considerations, well-documented protocols, and their ability to be easily manipulated. For example, cells can be transiently or stably transfected to introduce a gene of interest, enabling visualisation of a given protein using fluorescent protein tags. Cell lines offer an isolated monoculture of a single cell type, or co-culture of multiple cell types, which typically takes the form of a thin adherent monolayer or suspension culture [33,34]. For adherent cells, a uniform monolayer permits improved light penetration for imaging and consistent staining/immunolabelling, without the need to permeate dyes and antibodies into deeper layers of a sample. This reduces sample-sample variability, providing more reproducible imaging results. Moreover, cultured cells exhibit less endogenous fluorescence, which can interfere with label detection, compared to processed tissues and organs [35], and do not require time-consuming tissue processing for imaging. A microscope equipped with an incubation system enables real-time visualisation of live cells [36] and time-lapse imaging [19], with minimal interference to normal cellular function due to environmental disturbance.

Microscopy using cell line models enables the exploration of subcellular processes that are implicated in disease pathogenesis, as well as cellular responses to therapeutics [37,38,39]. Whole cell imaging of two-dimensional (2D) cultures allows the examination of cell morphology [40] and intercellular communication networks, mediated by structures such as filopodia or cytonemes [41], tunnelling nanotubes [42], and cellular bridges [43]. Live cell imaging of cell cultures also enables the study of dynamic cell behaviour, such as extracellular vesicle formation, cell motility and migration in wound healing [44], and cancer metastasis [38]. Fluorescence microscopy of cell culture is also widely used to image subcellular components, including organelles and molecules, to provide greater insight into their structure, function, and subcellular localisation. Recent examples of the impressive details of cellular structures visualised by super-resolution microscopy (SRM) include nuclear pore complex organisation, membrane-associated periodic skeleton in neurons and synaptic structures [45]. Multispectral imaging, using confocal and lattice light-microscopy, revealed an intricate spatial-temporal organelle interactome in live cells [46], illustrating the usefulness of novel microscopy techniques to better understand complex cell biology.

Despite the benefits of cell culture models, several limitations need to be considered when examining cells by fluorescence microscopy. Live cell imaging, especially for prolonged time periods, requires the maintenance of viable cells under appropriate growth and culture conditions. Culture media are multicomponent solutions, containing various micro and macro elements, vitamins, proteins, fatty acids, and pH indicators, which can themselves be fluorescent or can interfere with the fluorescent properties of a given fluorophore [47]. For example, phenol red, present in most culture media, is fluorescent when excited at ~400 nm [47,48] and some vitamins (e.g., riboflavin and pyridoxal) increase photobleaching of GFP [49], while serum albumin can bind to some fluorescent probes to alter their spectral properties [50,51]. Concentrations of dyes also need to be carefully optimised to ensure specific staining of target structures or molecules. In addition, cells may respond to the fluorescent compounds, introducing artefacts that need to be considered and accounted for with appropriate controls. Likewise, the overexpression of fluorescently tagged proteins via transfection introduces artefacts into these cell models. As with all biological samples, cell cultures are highly susceptible to phototoxicity, and establishment of appropriate nontoxic detection settings is critical. With well-planned experimental approaches and appropriate controls, fluorescent imaging of cell cultures can be relatively simple and reproducible, providing a range of possibilities and techniques to study different processes and events at the cellular and subcellular level. Thus, the most significant drawback of this system is that cell culture models may fail to replicate in vivo biology, since they lack the complex network of interactions or intercellular signalling, which occur in tissues, organs, or whole organisms.

### 3.2. 3D Cell Cultures

The need for animal-free disease models that avoid the limitations of traditional cell culture monolayers, yet better mimic human in vivo environments and recapitulate the complex interactions between different cell types, has resulted in advances in 3D cell culture models. These can represent many tissues, including brain [52], breast [53], and prostate tissue [54], utilising scaffold or scaffold-free techniques to induce or cultivate their formation or maintenance. There are multiple types of 3D cultures, ranging from spheroids of cell lines to organoids derived from patient tissue (reviewed by Caleb and Yong [55]). Indeed, patient-derived organoids are utilised to offer personalised treatments and novel therapeutic discoveries (e.g., [56,57]). Generated from cell lines cultures, spheroids or organoids may preserve the cell phenotypes observed in tissues, which result from interactions and responses to the microenvironment, such as detecting changes to necrotic tissue due to nutrient starvation in the centre of spheroids, mimicking rapidly growing non-angiogenic tumour tissue [58]. An example of the differences between 2D and 3D culture was demonstrated in cardiac cells; cells grown in 2D exhibited a large network of microfilaments and microtubules, whilst cells in a 3D environment were smaller in size, had many junctions between cells, and exhibited increased alpha actinin cytoskeletal protein [59]. Thus, 3D cell cultures may provide significant new insights into more complex cell biology, as well as disease biomarkers and therapeutics to improve translation.

Combining 3D cultures with 3D imaging is now being used to trace tissue development by stem cell differentiation and to determine responses to therapeutics. Recently, using a combination of immunohistochemistry and fluorescence microscopy, subpopulations of cancer stem cells were revealed to be affected in organoids via modulation of Wnt signalling upon Tankyrase inhibition [60]. These experiments were performed using morphometric analysis, with DAPI and F-actin fluorescence, to determine that the features that best described the compound-induced morphological changes are total organoid size, shape and size of lumen, live/dead cell counts, and changes in nucleus morphology [60]. Spheroid-derived image data are then used to produce characterisation methods that estimate growth and biophysical characteristics of multicellular tumour spheroids [61]. Using these spheroids, changes in cellular density and mechanical interactions are characterised, helping estimate cell diffusion, proliferation, and traction forces exerted by cells on the surrounding extracellular matrix [61,62]. The development of such protocols will be of significant benefit to the use of 3D culture in studying responses to therapeutics; however, there remains a need to investigate spheroids at a subcellular level to elucidate phenotypic changes occurring due to cell–cell interactions. Three-dimensional models have limitations for studying cell biology, as they still do not fully represent in vivo biology and, consequently, are still in development; as are the approaches to imaging these systems.

The ability to discover cell phenotypes and changes in disease processes, such as altered cell junctions, requires the use of fluorescence techniques to visualise subcellular protein location, expression, and distribution detectable by confocal microscopy. For example, trophoblastic cell spheroids have been utilised to investigate intracellular organisation and ciliary markers in cells starved of serum or treated with cytokines [63]. The accumulation of lipids and other light-scattering agents in multiple layers of cells within tissue presents a major hurdle to visualise the same biology in spheroids and organoids. Studies investigating extracellular matrix composition in spheroids [64] and evaluation of tight junctions in human intestinal organoids, have used cryosections [65] and immunohistochemistry to define the architecture and distribution of multiple markers (e.g., colorectal cancer [66] and corneal limbal organoids [67]). By performing serial sections, the structure may be re-created to reveal significant information about tissue; however, efficiency may be increased if antibody labelling and imaging could be performed on a minimally processed, intact organoid. To overcome the opacity of multiple cell layers, clearing methods can be used to render fixed spheroids and organoids transparent, through matching refractive index (RI) of the sample to increase transparency and allow more light penetration and reduced refraction. RI mismatches change the speed and angle penetration of light propagation onto cells, promoting light scattering, dispersion, and increased opacity [68]. For example, cell lipids and proteins have an RI value of ~1.44, whilst cytoplasm and nuclei have an RI of ~1.35 [69]. Hence, there are several optical clearing methods that aim to match the RI of the sample, increase sample transparency, and decrease light scattering to improve light penetration, imaging depth, and contrast [70]. Previously, these methodologies required long incubations of tissue, such as brain, for up to two weeks [71] or in toxic solvents [72]. However, new methods are being developed that are suitable for clearing spheroids more rapidly for higher-throughput protocols [73,74]. Our own analysis on prostate cancer spheroids has used a modified protocol from Ineveld et al. [74], and a combination of urea, fructose, and glycerol, to image beyond 100 µm deep in spheroids, at a sub-micron pixel resolution. These improvements are, thus, likely to expand the use of 3D cultures for applications using fluorescence microscopy to study more detailed cell biology.

As light-sheet microscopy and multiphoton microscopy become more widespread, these techniques are likely to be the imaging options of choice for 3D cultures, due to their significantly improved imaging speed, light penetration, and reduced photodamage, compared to confocal microscopy [75,76]. These techniques also offer a significant advantage in that (in theory) commonly used fluorescence tools and protocols are applicable. Unfortunately, in practice, the application of many existing fluorescently labelled antibodies and dyes can be challenging to adapt to 3D cell culture systems, due to poor penetration of the often bulky molecules through multiple cell layers [76]. Transgenic cell lines containing fluorescent proteins can overcome this issue; however, this is not applicable when working with organoids grown from primary cells. As the use of organoids and 3D cultures becomes more common, we are likely to witness improved staining protocols and products to address these current challenges. The combined use of cleared and fixed organoids for high-resolution 3D analysis without the requirement of immunohistochemistry techniques, together with new label-free live cell imaging and 3D culture, is likely to become the standard for cell biology analysis in the near future.

### 3.3. Tissue Sections

A truer representation of in vivo biology is of course to take it directly from the source: tissue. The major advantage of using tissue sections compared to cell line models is that the complex interactions between a cell with its microenvironment are preserved, including the presence of the supporting extracellular matrix [77], the influence of stroma and immune cells [78], and the maintenance of cell polarity in the hierarchical architecture of the tissue [79]. The ability to collect a snapshot of in vivo biology and preserve this for future study is invaluable, and biobanks with archived tissue exist for a multitude of diseases.

Tissue samples can be sourced in two forms: formalin-fixed paraffin-embedded (FFPE) or frozen. FFPE tissue is the most common and accessible sample type, with tissue preserved by formaldehyde fixation and embedded in paraffin wax blocks. In this way, large tissue collections can be routinely processed, and easily stored at room temperature to remain viable for decades. Compared to FFPE samples, cryopreserved frozen tissue is a much more limited resource but has several unique applications. ‘Fresh’ frozen tissue is generally snap-frozen in liquid nitrogen or isopentane immediately after resection, while ‘fixed’ frozen tissue is either perfused with fixative before cryopreservation to better retain tissue morphology and protein antigenicity, or fixed after snap-freezing for downstream application. Tissue can be a relatively scarce and precious resource, especially human tissue, which needs to be either sourced from biobanks, collected post-surgical removal, or obtained from animal models for disease. In all cases, gaining access to human or animal tissue can often be a costly and time-consuming endeavour, which is why cell culture can be an attractive alternative despite its discussed drawbacks. However, the ability to study tissue with all its complexity is invaluable and well worth the exertion.

Protocols for many applications exist for use on both frozen and FFPE tissue sections, and the choice of sample is a compromise between many factors. FPPE tissue is routinely collected in diagnostic labs and the process of embedding in paraffin maintains the integrity of the tissue architecture, making FFPE sections superior for morphological studies. However, non-standardised protocols between different institutions in tissue handling, fixation, and processing can create significant variability between samples with regards to background staining and analyte intensity [80]. A major concern with using tissue sections for fluorescence microscopy is inherent autofluorescence in the sample. Several components of tissue auto-fluoresce, including collagen [81], elastin [82], red blood cells, and some immune cells [35]. FFPE sections are more susceptible to this compared to frozen tissue, exacerbated by artefacts carried over from suboptimal fixative procedures or choice of fixative [83]. In frozen samples, tissue constituents such as lipids, which are removed in most standard FFPE processing protocols [84], proteins, and nucleic acids, are preserved in their native states. FFPE tissue processing creates extensive cross-linkage of proteins, which may mask some protein antigens in their native state [85]. This makes frozen tissue useful for molecular analysis such as DNA sequencing [86] and study of post-translation protein modifications compared to FFPE in which these may be poorly preserved. This can be particularly advantageous for fluorescence in situ hybridisation (FISH), which uses a fluorescent probe to label and spatially map nucleic acids in tissue sections [87]. However, during the freezing process, ice formation within the sample can damage tissue morphology and this can create artefact with distortion of the tissue compromising morphological characteristics. These varying factors need to be carefully considered for each experimental application and what level of sample quality is required, for which there are many useful reviews discussing tissue artefact and comparing handling protocols [88,89,90].

Although imaging of both 3D cell culture and tissue sections are widespread in investigating cell biology, to date, most biological research relies on standard cell culture and ex vivo models that cannot fully recapitulate physiological conditions, sometimes leading to artefacts and inaccurate results [91,92]. Imaging of biological processes in the context of a living organism opens new avenues in biomedical research, allowing visualisation of cellular and molecular associations in real time in their natural environment. Intravital or in vivo fluorescent microscopy has become a quintessential tool in the direct visualisation of the biological processes in living animals, with significant applications in studying alterations in tissue morphology and function [93,94], redox dynamics [95], cell proliferation and differentiation [96,97], cell migratory behaviour [98,99], tumour microenvironment [100,101] reviewed in [102], intracellular ionic activity [103,104], and host–pathogen interactions [105].

### 3.4. Intravital Imaging

Intravital imaging requires access to a high-resolution confocal, multiphoton, and/or light-sheet microscope, and the development of suitable animal models. Transgenic animals modified to express fluorescently tagged proteins provide a true in vivo environment for evaluating various cellular processes, with constitutive replenishment of fluorescently labelled proteins enabling long-term tracing in a cell- and tissue-specific manner. This technique mainly makes use of small organisms, such as fruit flies [106,107,108], zebrafish [95,109,110], and mice [111,112], due to their short generation times, established genetic lines, and relatively low cost. Subcutaneous and orthotopic xenograft models used in intravital studies can accurately provide insights into tumour heterogeneity and responses to drug treatments [113,114]. With the use of intravital microscopy, it has also become possible to visualise the inter-individual variability at a microscopic level in response to drug treatment. An advantage of intravital imaging is the continual monitoring of physiological changes over days and weeks, which is especially important in developmental cells [97], stem cell [115,116], and tumour biology [117]. Moreover, longitudinal intravital imaging can be performed with fluorescently labelled antibodies and dyes that can be intravenously injected for the simultaneous visualisation of, for example, individual lipid droplets and microvasculature in the liver [118], or even vessels in the femoral and calvarial marrow [119]. Recent advances in optics have brought intravital imaging into a new era of high-resolution fluorescence microscopy. These include digital adaptive optics scanning light-field mutual iterative tomography (DAOSLMIT) that utilises computational imaging from tiled wavefront correction with high-speed and high-resolution multi-dimensional imaging with low phototoxicity [120], to enable longer and higher-speed 3D subcellular imaging, to better study intercellular and intracellular interactions in tissue. Likewise, FLIM-FRET imaging has recently been applied to intravital imaging of kidney to study metabolic changes in different cell types through the organ [121].

Although intravital imaging is a powerful tool to study various biological processes in live organs and animals, several challenges need to be addressed, including anatomical constrains (i.e., organ accessibility), selection of appropriate techniques (e.g., type of incision and immobilisation of organ), and the use of inverted/upright microscopes (reviewed in [122]). Long-term imaging at a single-cell resolution in the cornea, skin, and hair follicles [123,124] does not require special preparation for obtaining optical access; however, small animals such as *Drosophila melanogaster* and zebrafish need to be anesthetised prior to imaging to permit the required surgical procedures to expose specific organs [106,109]. Accessing other organs of interest in larger animals such as mice is more challenging; the organ of interest can be surgically exposed, preserving its structure, connectivity, and perfusion in the anesthetised animal [125,126]. This invasive procedure may alter physiological homeostasis and can only be used for short-term imaging without impacting negatively on data for easily accessible sites, such as dermal vasculature [127], popliteal lymph nodes [128], salivary glands [126], small intestine [129], and liver [130]. Methods for imaging heart, kidneys, pancreas, and spleen are less developed and are at greater risk of artefacts that may result from inflammatory reactions. Long-term imaging of cellular processes requires installation of the optical windows adjacent to the organ of interest in a live anesthetised animal (reviewed in [131,132,133]), which permit unlimited microscope access to various abdominal organs [97,134], lungs [135,136], tumour-associated vasculature [137] and lymphatic vessels [138], spinal cord [139], and brain [140]. Although this technique allows visualisation of tissues/organs in their orthotopic location, implantation of the windows can lead to infections and motion artefacts. Moreover, animals can die during the surgery and the optical window can break or detach after successful implantation. These problems can be overcome by the use of optical fibres that provide access to sites deeper within the organ [141,142], or with micro-endoscopic probes employed to provide a less invasive observation mode of cellular-level features in colon [143,144], oesophagus [145,146], and trachea [147]. Fluorescent micro-endoscopy is a powerful technique for imaging the internal structure of a hollow organ; however, it can have a lower resolution and smaller field of view compared to high-resolution microscopes used in the techniques above. Furthermore, the physiological motion of the animal through respiration and cardiac activity can introduce significant imaging artefacts and compromise spatial information, particularly if the raw acquisition data remain uncorrected by special image processing algorithms [148,149].

Techniques and methodologies for intravital imaging are somewhat further advanced than those developed so far for 3D cell culture imaging. However, the methodologies still require expert knowledge and equipment to maintain animals during experimentation. In addition, ethical considerations need to be made when using these approaches, which go beyond those of standard animal experiments. Fluorescence microscopy of preserved tissue or organism post-mortem, therefore, is a more common approach when working with animal models. The challenges encountered when using fixed tissues or whole organisms mostly relate to sample thickness and fixation artefacts. As discussed above, tissue significantly hampers light and antibody/dye penetration, and requires further sample preparation, including sectioning and optical tissue clearing. The major drawback of imaging tissue post-mortem, when compared to intravital imaging, is the loss of dynamic processes and introduction of potential artefacts by surgical excision of the organ of interest and fixation processes. However, given the extensive use of animal models for biomedical research, the fluorescence microscopy of fixed animal tissues is widely used and has contributed significantly to our understanding of tissue morphology and molecular dynamics under normal and disease conditions.

## 4. Imaging Agents Used for Fluorescence Microscopy

Fluorescent labels are pivotal for fluorescence microscopy, allowing the detection and monitoring of a range of cell types and subcellular structures, which do not naturally fluoresce. Imaging agents are ideally water soluble and non-toxic species, which exhibit favourable photophysical properties, including large Stokes shifts, high quantum yields, resistance to photobleaching, long lifetimes, and excitation/emission properties amenable to commercially available hardware. There are four main categories of imaging agents for fluorescence microscopy applications: fluorescent proteins (FPs), graphene quantum dots (GQDs), metal-ion complexes, and organic fluorophores. Each of these classes have their own set of advantages, limitations, and experimental considerations, as discussed below. Although other types of imaging probes exist, such as radio-labelled contrast agents [150,151,152] and nanoparticle formulations [153,154], we have not included these here, as they are more frequently used in imaging techniques outside of fluorescence microscopy, such as magnetic resonance imaging (MRI) [155], positron emission tomography (PET) [156], computed tomography (CT) [157], single-photon emission CT (SPECT) [158], surface-enhanced Raman scattering (SERS) [159,160], and planar scintigraphy [161]. The following sections are designed to provide a brief understanding of the outlined four categories, with the selected examples demonstrating their use in fluorescence microscopy and the biological system/hardware conditions employed. This section also highlights the need for effective collaborative interactions between chemists, biologists, and physicists to solve critical problems in fluorescence imaging.

### 4.1. Fluorescent Proteins

The discovery of GFP in 1962 [162], and its subsequent cloning 30 years later [4], has led to FPs becoming an integral tool for cell biologists to monitor cellular processes using fluorescence microscopy. Since that time, a variety of FPs have been synthesised, which traverse the visible spectrum and have been well covered in some excellent reviews [163,164]. Fluorescent proteins are large molecules (typically 25–30 kDa) that exhibit bright fluorescence and excellent bioavailability [163]. The main point of difference between FPs and the other imaging strategies reviewed below is how they are applied. Typically, the FP is introduced into a cell via genetic encoding to express as a fusion tag to a protein of interest, as opposed to applying an exogenous labelling agent. Transfection obviates any cellular penetration issues, which can be experienced by other probe types [165]. Furthermore, this approach inherently allows for quantitative imaging as FPs can be expressed in a 1:1 ratio with their target substrate [166].

The impact that genome coding can have on the normal operation of the cell should always be a consideration. For example, cells transfected with enhanced GFP (eGFP) have been shown to trigger the proliferation of harmful reactive oxygen species [167]. The large size of FPs also need to be considered with respect to perturbation of normal functioning of the endogenous protein [168] and the over-expression of FPs, which can lead to aggregate formation [169,170]. Inaccurate interpretation of protein distribution and function resulting from these issues should be carefully controlled for in the experimental design. The robustness of FPs can vary considerably with construct purity, labelling [171], and excessive background fluorescence [172]. Typically, FPs are unable to image acidic environments [173,174]; however, examples do exist where this property has been exploited. For example, Trejo and co-workers used a pH-sensitive GFP (pHluorin-mKate2) to monitor starvation-induced autophagy in LC3B transgenic mice, where the FP was non-fluorescent in acidic compartments, but was emissive in neutral or basic environments [175].

A major disadvantage of FPs is their high background fluorescence, which can make the processing of images difficult. To circumvent this issue, FPs have been designed where a non-fluorescent protein becomes emissive upon activation by a trigger [176]. A recent example from Wu et al. reported the use of FPs, which bind to RNA aptamers to evoke a “turn-on” fluorescent response and allow visualisation of mRNA in live HEK293 cells using an epifluorescence inverted microscope [177]. This approach significantly improved background fluorescence when compared to the more traditional MS2-MS2 coat protein (MCP) tethering approach used to image mRNA [178,179,180]. Other examples have adopted this approach to image RNA trafficking [181,182].

**Figure 2:**
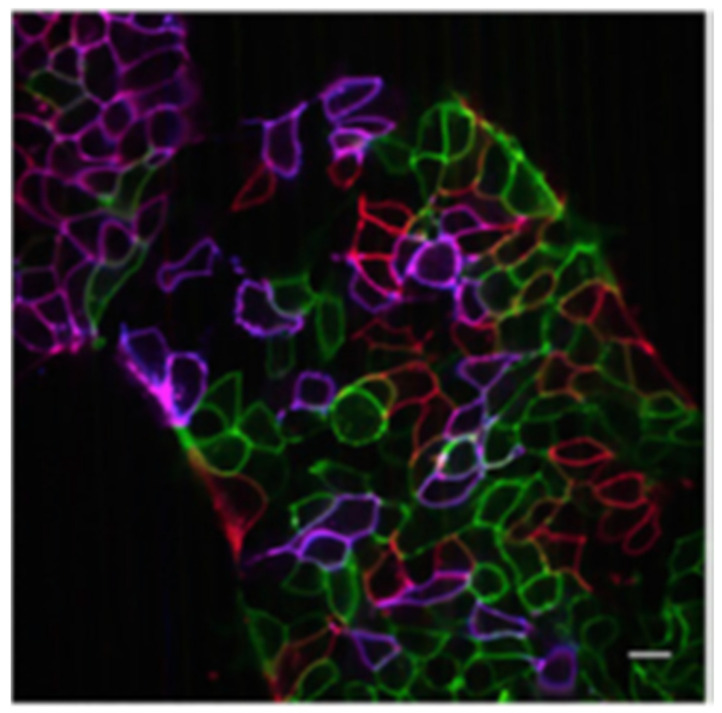
Live human-induced pluripotent stem cells after 12 h with transfected with eGFP, mOrange2 and mKate2. Scale bar = 20 µm. Reproduced with permission [185]. Copyright 2020 Elsevier.

Simultaneous expression of several FP markers allows multicolour labelling of cellular process, compartments, or proteins of interest to be investigated in parallel within a cell. The “Brainbow” strategy relies on a handful of spectroscopically distinct FPs to provide information of different cellular types and environments, by emitting a broad palette of detectable hues [183]. Hematopoietic stem cells, which give rise to B and T cells, have recently been imaged by transplanting a range of fluorescently labelled cells with differing emission profiles, to provide information on hematopoietic cell lineage in Rag1^−/−^ mice using confocal microscopy [184]. Similarly, live pluripotent stem cells were imaged by confocal microscopy to track their eventual derivatisation using the Brainbow technique (Figure 2) [185]. This approach has recently been supplemented with a secondary near-infra red FP (mCardinal), whose expression is driven independently of the Brainbow cassette [186]. This novel combinatorial system opens the door to further expand the Brainbow and related multi-colour techniques of FPs.

**Figure 3:**
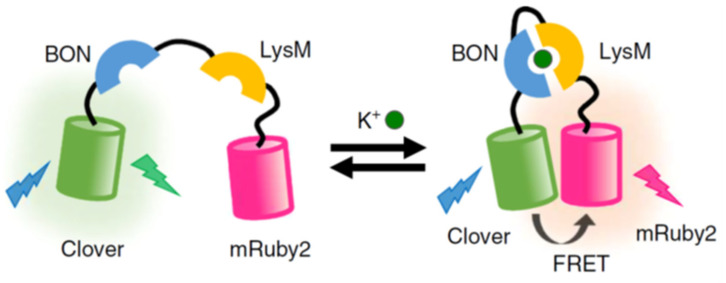
Schematic of the KIRIN1-GR FRET system where emission at a higher wavelength is detected after potassium binding. Image adapted with permission [189]. Copyright 2019 Springer Nature.

Despite the considerable effort to construct new FPs with different optical parameters, the field is still largely limited by spectral overlap between their broad fluorescence profiles [187,188]. This characteristic of fluorophores is taken advantage of in applications such as FRET. A recent example of a FP-based FRET system was reported by Shen et al., who created three independent potassium ion (K^+^) sensors to measure both intra and extracellular K^+^ [189]; a critical electrolyte for cell function [190]. This was achieved in live HeLa cells using confocal microscopy. The overall design of each system (Figure 3) relied on a “turn-on” effect when two potassium binding proteins encapsulated K^+^, which brought the FRET donor and acceptor FPs close enough to elicit a FRET response. FRET sensing is also widely used to monitor protease activity by coding FPs linked together by a protease-specific cleavable site, leading to a “turn off” effect. This strategy has been used to measure common proteases such as Caspase-3 [191,192], Trypsin [193,194], Thrombin [195,196], and Calpain [197,198]. FRET-based systems continue to be an area of interest to extend emission into the near-IR part of the visible spectrum, by tailoring FP donor and acceptor photophysical properties [199,200]. The main advantage of using FPs for FRET imaging is that the fluorescent donor and acceptor are produced by the cells, which obviates the need for small molecule labelling and issues encountered with cellular penetration. Excluding these specialised uses, overlapping excitation-emission profiles remain an issue when using FPs, and a judicious selection of fluorophores is required to maximise photophysical potential of the system and to limit unwanted behaviour alterations of the fusion protein from coding in the FP genes [171,173,201].

The ability of fluorescent proteins to be genetically encoded into a cell as a non-invasive fluorescent tag, without the need for fixing or permeabilising cells, makes them indispensable for live cell imaging, and may be increasingly useful in 4D imaging of opaque samples such as spheroids [202], where the penetration depth of antibodies and dyes may be limited. Imaging in four dimensions can shed light on intracellular dynamics, such as protein trafficking and organelle interplay [203], and has also been applied to small single-celled systems such as yeast [204]. For example, fast 3D-phase imaging combined with 3D super-fluorescence microscopy has allowed the imaging of actin filaments in live RAW 264.7 macrophages transfected with the Lifeact-Dreiklang FP [205]. Another recent example uses fluorescence correlation spectroscopy to generate high-throughput quantitative 4D images for a range of counting applications; this method can be used with effectively any monomeric FP such as mCherry and mScarlet [206]. The advantages of using FPs for 4D imaging is proving to be a hot topic, and we expect significant growth in this area as the technology continues to be developed.

### 4.2. Graphene Quantum Dots

Graphene quantum dots (GQDs) are zero-dimensional fluorescent nanomaterials that have been exploited for a variety of imaging and sensing applications. This category of imaging technology is the most recent of the classes reviewed here; however, their potential for greater use in fluorescence microscopy is significant with a surge of recent examples within the literature. We have focussed on GQDs rather than other varieties of inorganic QDs, which often suffer from solubility and toxicity issues owing to their reliance on incorporated heavy metals such as cadmium, mercury, and lead [207,208]. Conversely, GQDs are water soluble materials which have excellent toxicity profiles [209,210,211]. The photophysical properties of GQDs are amenable to fluorescence microscopy with higher molar absorptivity coefficients, improved photostability and longer-lived emission compared to organic fluorophores [208]. A major advantage of using GQDs is how easily they can be synthesised using either a ‘top-down’ or ‘bottom-up’ approach [212,213,214]. Of these two strategies, the ‘bottom-up’ approach has become the favoured route, given starting material affordability and the simplicity of the method, which does not require specialist chemistry or cellular biology training, unlike other imaging strategies reviewed here.

A drawback of using GQDs is that by virtue of their synthesis, a heterogenous and inseparable mixture of chemical structures is formed, which inherently makes characterisation difficult when using traditional analytical techniques employed for small molecules, such as NMR spectroscopy and mass spectrometry [215]. As a result, techniques such as IR, XPS, and UV-visible spectrophotometry are relied on to characterise the bulk material. This structural ambiguity means that functionalisation of the GQD surface is also more difficult when compared to organic fluorophores, as the exact number (and sometimes type) of reactive handles are unknown and reaction monitoring is almost impossible. Nonetheless, advancements have been made with several successful functionalisation strategies having been summarised in review articles [215,216].

**Figure 4:**
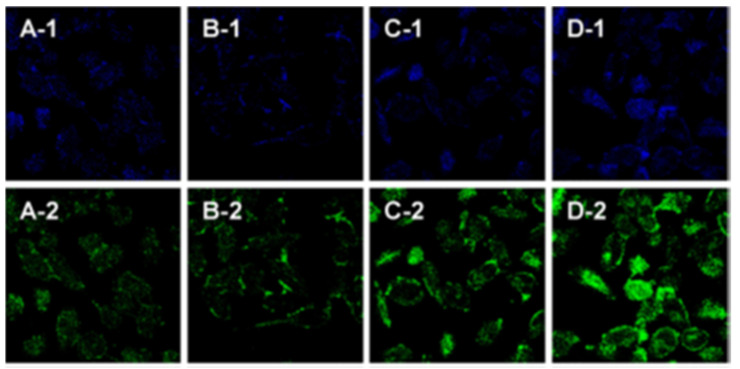
Confocal micrographs of HeLa cells incubated with 1 mg mL^−1^ folic acid-encapsulated GQDs for 6 h at various pH (5, 6, 7, and 8; **A**, **B**, **C**, and **D** respectively). **1** (425–490 nm, λ_ex_ 405 nm) and **2** (500–570 nm, λ_ex_ 488 nm) refer to the different spectral windows. Reproduced with permission [220]. Copyright 2018 Elsevier.

Initially, GQDs were explored for their viability as cellular imaging agents by evaluating toxicity, cellular uptake, and visualisation on a range of cells [217,218,219]. Once it was apparent that these materials were amenable to such endeavours, more sophisticated systems were designed. For instance, pH sensing in HeLa cells using folic acid-encapsulated GQDs has been reported, where a pH increase from 5–8 results in an increase in emission intensity in the green detection window (500–570 nm, λ_ex_ 488 nm), using confocal microscopy, while the emission intensity in the blue detection window (425–490 nm, λ_ex_ 405 nm) remained relatively unchanged (Figure 4) [220]. A more recent example has used nitrogen-doped GQDs to detect temperature in live HeLa cells by measuring the temperature-dependent fluorescence quenching as the cells are warmed from 25–45 °C [221].

**Figure 5:**
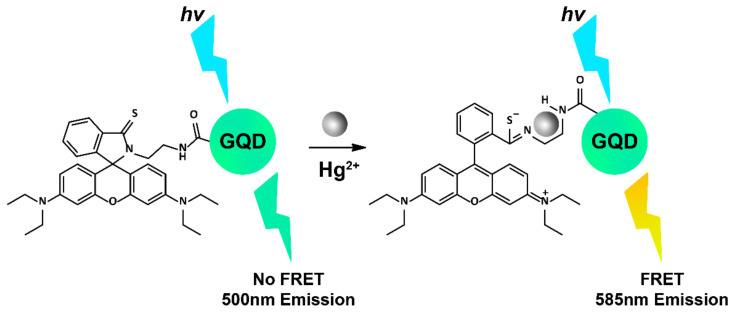
Proposed FRET mechanism of rhodamine-GQDs detecting Hg^2+^. Image adapted with permission [224]. Copyright 2015 Elsevier.

The detection of mercuric ions (Hg^2+^) in living cells is an important pursuit given the high toxicity they impart on the body, which can lead to kidney, liver, and brain damage [222]. GQDs coated with thymine-rich DNA have been reported to accurately detect Hg^2+^ in HeLa cells, whereby Hg^2+^ strongly coordinates with thymine residues to create a rigid hairpin on the GQD surface, ultimately decreasing the GQD emission at 460 nm by approximately 90%, as observed using confocal microscopy [223]. Another example of intracellular Hg^2+^ detection utilised GQDs conjugated with rhodamine fluorophores to create a FRET ‘turn-on’ system in the presence of Hg^2+^ (Figure 5). The FRET construct demonstrated excellent reversibility (up to five cycles) and good biocompatibility in HeLa cells [224]. Other metals have been successfully detected in live cells using GQDs too, including a ratiometric GQD-Nile Blue conjugate for two-photon detection of Cu^2+^ ions in A549 cells [225] and nitrogen-doped GQDs, which could reversibly detect Al^3+^ and had demonstrated imaging capacity in HeLa cells [226]. The sensing of non-metal ions critical in biological systems has also been achieved using GQDs. For instance, nitrogen-doped GQDs were used to detect nitrite (NO_2_^−^) as low as 2.5 nM in live human bladder carcinoma T24 cells, using laser scanning confocal microscopy, with no cytotoxicity and good biocompatibility observed [227]. Hypochlorite (ClO^−^) is a vital ROS species endogenously produced in living organisms from hydrogen peroxide (H_2_O_2_). GQD sensors capable of detecting both of these chemicals in cells have been reported, with GQDs furnished with *o*-phenylenediamine units capable of detecting ClO^−^ at 69 nM in MCF-7 cells [228], and a FRET-based system employing a GQD conjugated with boronate merocyanine chromophore-efficient H_2_O_2_ reporters in HeLa cells using two-photon excitation [229].

**Figure 6:**
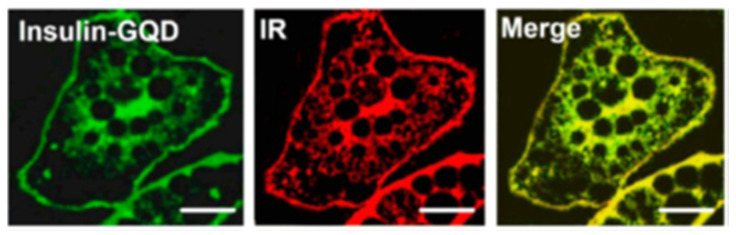
Confocal fluorescence images of fixed 3T3-L1 adipocytes. (**left**) Cells are stained with Insulin-GQDs; (**middle**) cells are treated with antibodies against insulin receptor B subunit followed by Atto647 NHS-conjugated secondary antibodies; (**right**) merged image to demonstrate insulin receptor staining. Scale bar = 10 µm. Reproduced with permission [232]. Copyright 2013 American Chemical Society.

Defining subcellular structures is a common goal in fluorescence microscopy and tools that allow this level of specificity are much sought after. Although this domain is mostly owned by organic fluorophores, GQDs have been utilised to track subcellular components too. For example, GQDs were functionalised with ethylenediamine groups (via an acid chloride route) to generate a probe which selectively stained the nucleolus in live HepG2 cells, as evidenced with co-staining experiments with the commercially available SYTO RNA-Select nucleolus stain [230]. The mitochondria has also been successfully targeted using a ratiometric GQD-cyanine conjugate FRET system, which is specific for mitochondrial peroxynitrite (ONOO^−^) and can detect ONOO^−^ at concentrations as low as 30 nM, as evidenced using laser scanning confocal microscopy on RAW264.7 cells [231]. Protein conjugation in order to monitor uptake and trafficking behaviour can also be studied using GQDs, as has been achieved with insulin-conjugated GQDs used to monitor insulin receptors in live 3T3-L1 adipocytes, confirmed by co-staining experiments (Figure 6) [232]. A similar concept was reported for tracking transferrin receptors with transferrin conjugated GQDs to monitor real-time imaging in HeLa cells [233].

Tools which can differentiate between cell and organ types are valuable in bioimaging applications. Although GQDs are still emerging as a viable option for these types of studies, there have been a handful of examples that clearly demonstrate their potential in this field. For example, in mice, near-infrared emitting GQDs were shown to preferentially localise in the kidney and liver, when compared to other organs [234]. A more sophisticated example has been recently published with Nd^3+^ and Tm^3+^ (rare earth metals)-doped GQDs used to image mice, with maximal uptake observed in the kidneys, liver, spleen, and intestine, using fluorescence microscopy [235]. Additionally, GQDs that exhibited emission in the yellow region of the visible spectrum showed efficient staining of stem cells when excited at 405 nm [236]. This type of work has naturally been extended into the theranostic and drug delivery space with GQDs finding use in targeting breast cancer [237], pancreatic cancer [238], and the delivery of Doxorubicin to HeLa cells [239]. For instance, GQDs decorated with hyaluronic acid moieties to enhance cancer cell uptake were used as drug carriers to deliver curcumin as a drug model to HeLa cells, where it was released upon exposure to the mildly acidic environment of the cancer cells. Consequently, this system imparts good selectivity with no toxic effect observed on normal cells [240].

### 4.3. Metal Ion Complexes

Metal ion complexes have been successfully used in cellular imaging applications such as subcellular compartment staining and visualisation of cellular processes. A variety of sensitising pathways have been utilised for these types of metal complexes, such as metal-to-ligand charge transfer (MLCT) and ligand-to-ligand charge transfer (LLCT), which have been reviewed previously [241]. The major advantage of using metal ion complexes as imaging agents are their long-lived emission profiles, which facilitate the use of time-gated fluorescence microscopy experiments, such as FLIM, and enables the visualisation of cellular events, which is otherwise not possible due to endogenous autofluorescence within cells [242]. Moreover, metal ion complexes offer highly tuneable excitation and emission profiles, which span the entire visible and near-IR spectrum. They also offer the largest Stokes shifts of the four classes covered in this review (typically greater than 5000 cm^−1^), which obviates self-quenching issues encountered by most organic fluorophores [243].

Metal ion complexes are still considered small molecules (when compared to much larger GQDs and FPs); however, their structures are typically more complex compared to organic fluorophores, which renders their synthesis more difficult. The metal core can often impart cytotoxic effects, which can limit their use in live cell imaging applications [244] and lead to poor water solubility making handling difficult. Despite these drawbacks, metal ion complexes have been successfully used for a range of live cellular imaging applications.

**Figure 7:**
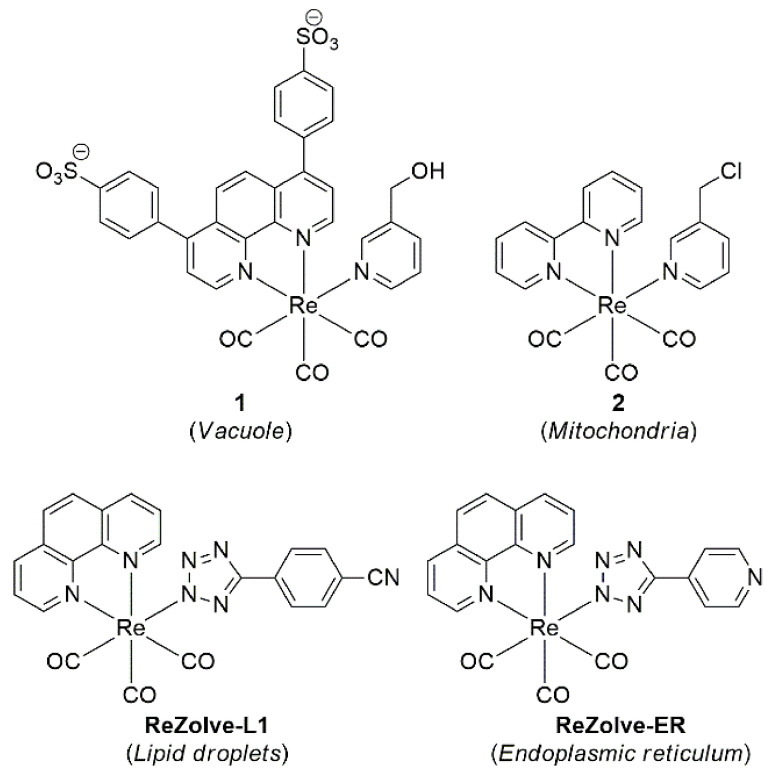
Reported Re(I) complexes with organelle localisation in parentheses.

Metal ion complexes for cellular imaging can be divided into two main sub-classes; low-spin d-block transition metals with d^6^, d^8^, or d^10^ electronic configurations, such as Re(I), Ru(II), Ir(III), and Pt(II), and f-block emissive lanthanoids, such as Ln(III), Eu(II), Gd(II), and Tb(III) [245]. Re(I) carbonyl complexes are the most common type of transition metal complex used in bioimaging [246], as they can be readily functionalised to serve a required purpose. For example, Amoroso and co-workers have reported two similar Re(I) tricarbonyl metal ion complexes with either hydroxymethyl or chloromethyl substituted pyridinyl ligands, which exhibit vacuole and mitochondrial localisation (**1** [247] and **2** [248] respectively, Figure 7). More recently, the Massi group has contributed to this area with a range of Re(I) complexes including **ReZolve-L1** featuring a benzonitrile group [249], which was the first report of a selective dye for polar lipids in live cells [250], and a pyridinyl substituted derivative (**ReZolve-ER**), which localises within the endoplasmic reticulum (ER), both evidenced using confocal microscopy [251].

**Figure 8:**
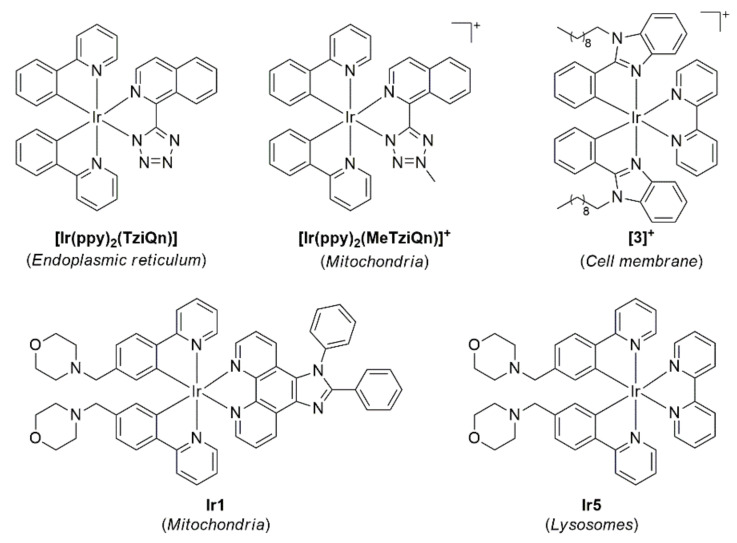
Structures of Ir(III) complexes with organelle localisation highlighted in parenthesis.

The Massi group have also conducted a thorough investigation of Ir(III) complexes and how a subtle structural change, from a neutral tetrazole group (**[Ir(ppy)_2_(TzIQn)]**, Figure 8) to a methylated cationic tetrazole (**[Ir(ppy)_2_(MeTziQn)]^+^**), can impart significant cellular uptake properties with H9c2 staining observed in the ER and mitochondria, respectively [252]. Interestingly, the cell uptake of these compounds was independent of lipophilicity. Conversely, another set of Ir(III) complexes have been reported whose toxicity and cellular uptake is strongly dependent on the lipophilic character of the probe (**3**, Figure 8) [253]; this is clear evidence that metal ion complexes need to be assessed on a case by case basis for cellular imaging capacity. Another example of how lipophilicity impacts cellular staining has been demonstrated using morpholino-conjugated Ir(III) complexes with varying LogP values. The morpholino group has been successfully used to target lysosomes for Ir(III) complexes in the past [254]; however, **Ir1** preferentially localised within the mitochondria (87% Pearson’s correlation coefficient with MitoTracker™), while the less hydrophobic **Ir5** was observed in the lysosome (79% Pearson’s correlation coefficient with LysoTracker™) [255]. These morpholino-conjugated Ir(III) complexes were further explored by adding a third morpholino group to improve water solubility and allow for two-photon excitation of long-lasting lysosome imaging in HeLa cells [256]. Other examples of Ir(III) complexes have been reported to sense changes in the mitochondrial environment, either for hypoxic conditions [257] or labile Zn^2+^ ions [258]. There are also examples of Pt(II) [259,260] and Ru(II) [261,262] metal ion complexes for cellular imaging; however, these are less favoured options when compared to the Re(II) and Ir(III) alternatives, presumably owing to their comparatively weaker photophysical properties. Moreover, transition metal complexes have been used for sensing purposes too, including endogenous nitric oxide [263] and thiol [264] sensing using Ru(II) complexes.

**Figure 9:**
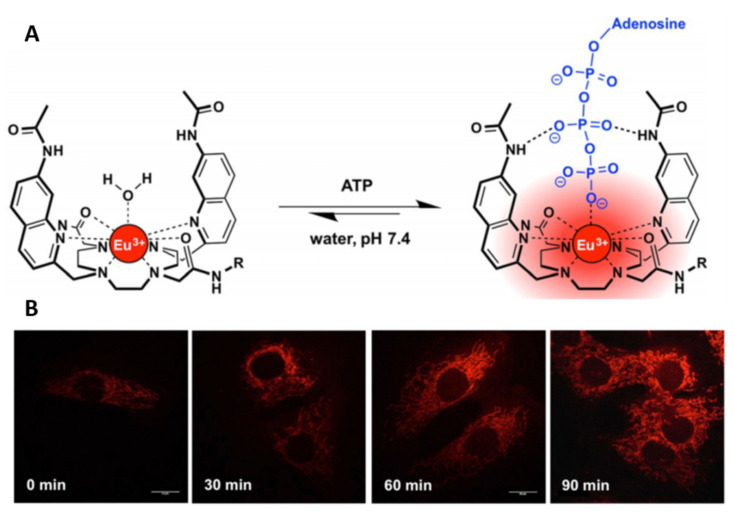
An Eu(III) complex for mitochondrial ATP sensing. (**A**) The proposed binding mechanism between an Eu(III) complex and ATP to elicit a ‘turn on’ luminescent response. (**B**) NIH-3T3 cells treated with staurosporine (10 nM) and stained with an Eu(III) complex (50 µM, λ_exc_ 355 nm, and λ_em_ 605–720 nm), demonstrating real-time monitoring of ATP levels in mitochondria. Images adapted with permission [268]. Copyright 2018 Wiley-VCH.

Trivalent metal ions from the lanthanide series of f-block elements have also shown extensive application for cellular imaging. These systems nearly always rely on an antennae group to populate the metal excited state, which gives rise to the characteristic phosphorescence signal exhibited by the lanthanide ion [243,265,266]. Additionally, these complexes require a chelator to affix the lanthanide ion in a position whereby energy transfer can take place. A wide range of ligand/chelator/lanthanide ion combinations have been reported and are well-reviewed [265,267]. Lanthanide complexes are normally used for the tracking of key biological processes or key biomolecules, rather than staining specific regions of the cell. For example, the concentration change of adenosine triphosphate (ATP) within the mitochondria has been measured effectively using cyclen-Eu(III) complexes, such as that shown in Figure 9. The quinoline arms of the complex likely aid in the Eu complexation and the pendant amide groups are proposed to aid in ATP binding. This was demonstrated in NIH-3T3 cells to provide real-time measurement of ATP in live cells by a ‘turn-on’ luminescent effect, using laser scanning confocal microscopy [268]. A similar design concept reports a lysosomal targeting Eu(III) complex for hypochlorous acid sensing in live RAW 264.7 cells, using time-gated luminescent imaging [269].

**Figure 10:**
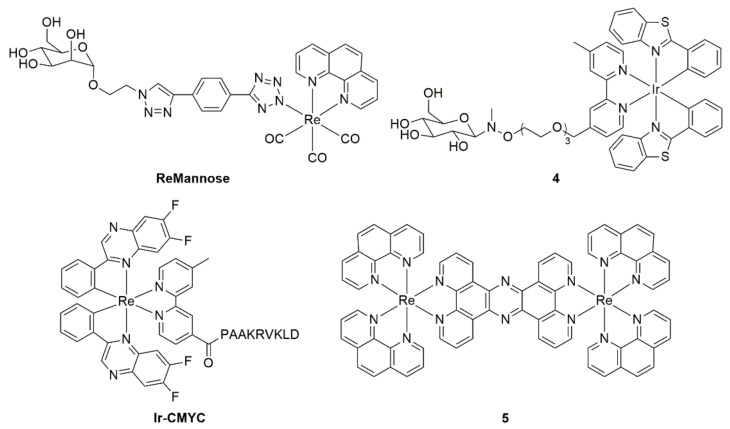
Structures of sugar conjugated metal ion complexes (ReMannose and **4**), nucleus imaging agent (Ir-CMYC), and DNA sensor (**5**).

The tracking of important biological molecules in live cells has also been achieved using metal ion complexes. Gillam and co-workers recently reported several neutral Re(I) complexes conjugated to sugar moieties (such as **ReMannose**, Figure 10) and tracked their biodistribution in H9c2 cells using confocal microscopy [270]. Interestingly, it was found that biodistribution was not directed by the sugar moiety and rather the larger Re(I) complex. Conversely, Ir(III) complex **4**, which contains a glucose moiety tethered by a polar TEG chain, exhibited cellular uptake through GLUT transporters in HeLa cells [271], suggesting that the increased spacer provided by the water soluble linker played a significant role in the uptake mechanism. Recent work by Day et al. has conjugated a signalling peptide (PAAKRVKLD) to an Ir(III) complex to drive nucleus uptake. This imaging agent (**Ir-CMYC**) demonstrated co-staining with commercially nuclear stain Hoechst 33342 in fibroblast cells, exhibited near-IR emission, and was non-toxic [272]. However, conjugation with the metabolite of interest is not always the method used to track intracellular processes. For instance, Gill and co-workers reported a dinuclear Ru(II) dimer (**5**), which interacts with DNA to provide an environment-dependent emission in both prokaryotic and eukaryotic cells. This probe does not contain any specific directing motif yet still exhibits good selectivity to DNA [273].

### 4.4. Organic Fluorophores

**Figure 11:**
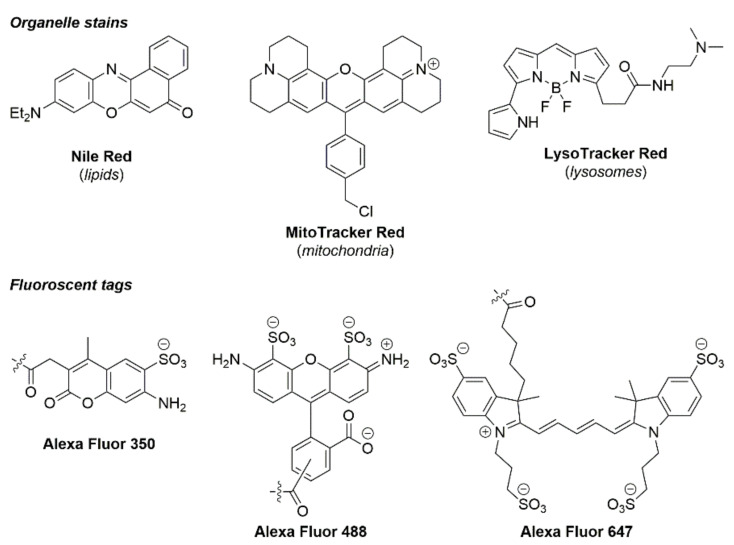
Commercially available fluorophores for organelle staining (**top**, target organelle in parenthesis) and bioconjugation (**below**).

Organic fluorophores enjoy a privileged place as the most prominent class of compounds for fluorescence imaging. Owing to their ready accessibility, small size, wide variety, and excellent emissive properties, organic fluorophores are frequently used for imaging cellular components, visualising cellular processes and tagging larger molecules (including antibodies and drugs) for insights into their cellular activity. The advantages and applications of organic fluorophores have been thoroughly covered in many excellent reviews [274,275,276,277,278], and their commercial availability renders their use as “plug and play” for cell biologists. Some common examples of commercially available fluorescent dyes include **Nile Red**, **MitoTracker™**, and **LysoTracker™**, for the staining of specific cellular organelles (see Figure 11), while the **Alexa Fluor** series of compounds are the most used tags for bioconjugation applications owing to their wide variety and established attachment chemistry. Structurally, these compounds are strikingly distinct and cover a range of different fluorophore classes; however, key functional groups are what typically drive cellular location for the organelle stains. For example, a diethylaniline is often used for lipid uptake (**Nile Red**), an overall positive charge typically leads to mitochondrial localisation (**MitoTracker™ Red**), and tertiary amines which become protonated in acidic environments are often employed for lysosomal uptake. In the case of fluorescent tags, the key functional group facilitates the actual linkage between the fluorophore and the target of choice. In the case of the Alexa Fluor series depicted in Figure 11, this is achieved using carboxylate groups, which enable the formation of amide bonds.

The chemistry of organic fluorophores has been well investigated and new classes continue to be reported in the literature. Design considerations to improve the internal charge transfer (ICT), quantum yield, lifetime, and cell viability are commonly considered by chemists synthesising novel probes. A major advantage of small organic fluorophores is their capacity to be attached to larger molecules for tagging purposes (for example **Alexa Fluor™ 350**, **488**, and **647**). However, organic fluorophores often suffer from short lifetimes, photobleaching, cell toxicity, and poor solubility in aqueous environments. Nonetheless, organic fluorophores remain an indispensable tool for fluorescence microscopists for a range of applications.

The most common use of organic fluorophores in cellular imaging has traditionally been tagging of larger biomolecules, such as antibodies, peptides, or therapeutics, to investigate the mode of action studies [279] and cell function [277]. The addition of a bulky imaging agent tethered to a molecule of interest inherently casts doubt on whether its endogenous activity will be impacted. The fact that organic fluorophores are normally low molecular weight compounds is a prime reason for their monopoly for tagging applications.

**Figure 12:**
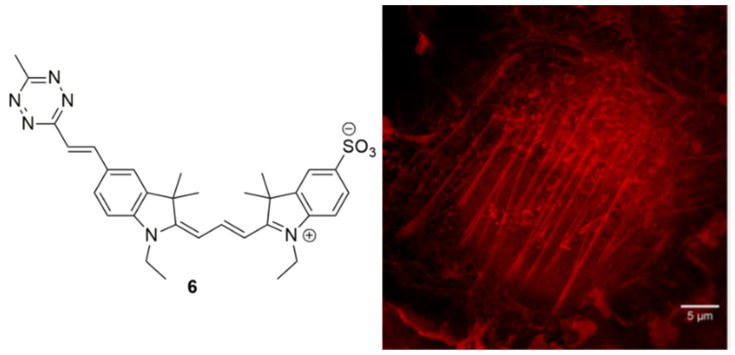
The structure of cyanine **6** and a micrograph demonstrating actin imaging in Cos7 cells after cyclisation with a small peptide. Scale bar = 5 µm. Reproduced with permission [280]. Copyright 2018 American Chemical Society.

The second most common use of organic fluorophores is to visualise subcellular structures. There are many commercially available dyes which are organelle-specific and are frequently used in co-localisation experiments to validate novel probes for this purpose, which may offer advantageous handling, photophysical, or toxicity properties. For example, a tetrazine-functionalised cyanine probe (**6**, Figure 12) has recently been used to react with cyclooctyne-labelled peptides to image actin filaments in Cos7 cells, using super resolution microscopy [280]. Lipid droplets (LDs) have garnered intense interest in the past few years as dynamic organelles essential to a wide variety of cellular processes. Imaging agents that selectively stain LDs are in high demand, such as the coumarin C-1, reported by Jana and co-workers, which was visualised in hepatoma cells at 200 nM, and confirmed using differential interference contrast (DIC) illumination [281]. An excellent example of how structural changes significantly impact subcellular localisation was illustrated by Collot et al., who reported three squaraine compounds, each with different structural motifs, ultimately leading to distinct staining patterns in HeLa cells [282].

**Figure 13:**
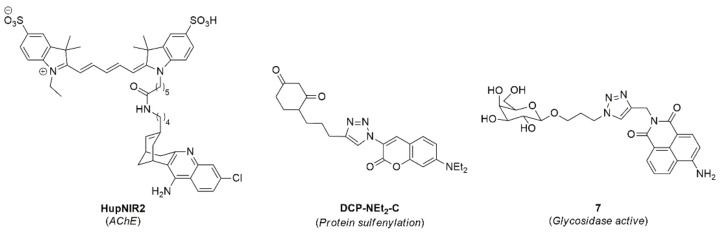
Organic fluorophores used for fluorescence imaging applications (shown in parentheses).

Organic fluorophores are also ideally suited to monitor important cellular processes, owing to their wide ranging and accessible chemistries, which allow the synthesis of chemosensors designed for specific applications. The function of acetylcholinesterase (AChE) is critical for the conversion of acetylcholine to choline and acetate; a vital component of the nervous system. Recently, two near-IR emitting cyanine probes were developed, which were conjugated to huprine derivatives to enable AChE binding. **HupNIR2** (Figure 13) demonstrated excellent binding efficiency to AChE (IC50 = 31 ± 1.5 nM) and was visualised using fluorescence microscopy in a range of different cell types [283]. Another example of important cellular functions being monitored using fluorescence microscopy was described by Holmila et al., who reported coumarin compound **DCP-NEt_2_C**, which was capable of capturing protein sulfenylation events, through reactivity with its 2,4-dioxocyclohexyl group, within the mitochondria of A549 cells, using confocal microscopy [284].

The ability of small organic fluorophores to act as drug-masks allows for the visualisation of drug delivery in real-time. For example, the Gunnlaugsson lab have reported a variety of chemosensors based on the 1,8-naphthalimide scaffold, including glycosylated derivatives (such as **7**) for the visualisation of glycosidase activity, as demonstrated in cancerous cell lines [285]. These probes were designed such that cellular uptake was only permitted after the glycosidase enzyme cleaved the glycan unit from the imaging agent. Organic fluorophores have also found use as effective therapeutic agents harnessing their absorptive properties to give rise to photo-dynamic therapy. For example, a cisplatin-BODIPY conjugate was recently reported which was found to initiate ROS upon irradiation with near-IR light to invoke a cytotoxic response to A549 and HeLa cancer cells [286]. This conjugate was found to localise within the mitochondria of cells, which is the major cite of ROS production. The use of organic fluorophores in theranostics and photo-dynamic therapies has been well covered in the literature and the reader is directed to some excellent reviews on these topics [287,288].

## 5. Conclusions

This review has briefly outlined the three fundamental factors that need to be considered when designing fluorescence microscopy experiments. The most financially expensive consideration is what hardware is available and whether the parameters of this instrumentation will be suitable for the desired result. Careful design of an appropriate biological model which is amenable to fluorescence microscopy is required. Finally, the most suitable imaging agent to provide the fluorescent signal needs to be selected, with an ever-growing range of commercially available and literature-reported examples on offer. These three considerations are not mutually exclusive, with each factor impacting on the other two and ultimately driven by the intended outcome of the experimental data required. Consequently, an understanding of all aspects of hardware, imaging compounds, and in vitro or in vivo models is important when designing fluorescence microscopy experiments. As highlighted by numerous examples here, confocal microscopy dominates as the fluorescence microscopy hardware setup of choice. This is likely due to accessible cost and availability of these microscopes and the broad applicability to a range of imaging probes and biological models. Greater uptake and more widespread use of the advanced imaging hardware discussed in this review is likely to accelerate in the near future, as biologists look to harness the power of these instruments to gain a deeper understanding of cellular mechanisms and biology. We believe that this review will be helpful for those not fully versed across fluorescence microscopy to steer them towards experimental conditions and parameters which will aid in generating high quality and informative images for their intended applications.

## Data Availability

Not applicable.

## Supplementary Materials

The following are available online at https://www.mdpi.com/article/10.3390/cells11010035/s1: A detailed comparison of different fluorescence microscopes (Table S1) and a general schematic describing fluorescence within a cell (Figure S1).
