# Supplementary material for: Fluorescence Microscopy—An Outline of Hardware, Biological Handling, and Fluorophore Considerations
Source: Cells. 2021 Dec 23;11(1):35. doi: 10.3390/cells11010035 (PMC8750338; doi:10.3390/cells11010035)
Supplement: Supplementary file 1 [file cells-11-00035-s001.zip › cells-1522549-supplementary.pdf]

Table S1: The table below provides a more in-depth description of fluorescence microscopes, along with their ideal applications, resolution limits and the advantages and limitations of using a particular microscope. In particular, the descriptions highlight if any special sample preparation techniques are required to make use of a given microscope. This table is non-exhaustive, and further reading can be accessed by the references cited.

Microscope Type	Application	Pros	Cons	Resolution Limit
**Wide Field Fluorescence**	Live cell imaging, tissue section imaging, multi well assays	Least expensive, easy to operate, multiple camera options, fast acquisition times	Limited light penetration, limited fixed filter combinations	< 200 nm @ λ_{ex} = 400 nm in air [1]
Wide field fluorescence microscopes present the most cost effective medium for fluorescence microscopy and are operationally simple with the least amount of componentry. There are many camera and excitation options, such as CCD (charge coupled devices), CMOS (complementary metal on semiconductor) cameras, and mercury, metal halide and LED (light emitting diode) excitation sources. As these light sources are relatively low intensity, light penetration into samples is limited. Wide field microscopes are also generally limited with fixed filter combinations, limiting the number of channels that can be imaged. Wide field fluorescence microscopes are ideal for quickly checking if transfection of cells has occurred or if fluorescent staining is positive.				
**Laser Scanning Confocal**	Live cell imaging, tissue section imaging, multi well assays, optical sectioning	Optical sectioning, high efficiency detectors, tuneable filters, highly configurable options	High cost of entry, relatively slow acquisition times	< 150 nm @ λ_{ex} = 400 nm in oil [2,3]
Wide field microscopes are limited in resolution and capture both in focus and out of focus light emitted from a sample. Laser scanning confocal microscopes can acquire high resolution images, as the out of focus light from the imaging plane is blocked by a pinhole. This allows optical sections of samples to be created, and in combination with z-stacking, allows for 3D models of samples to be generated. Confocal microscopes are typically suited for imaging sub cellular features. The excitation sources for confocal microscopes are usually solid-state lasers, which offer long usage lifetimes, but are limited to fixed excitation wavelengths. The emission filters of confocal microscopes are generally highly configurable and tuneable to specific ranges, allowing users to minimise crosstalk between fluorescence channels.<br><br>Laser scanning confocal microscopes are also offer the most flexibility in hardware configurations, with many applications such as FRET, FRAP and FLIM able to be added onto a basic confocal system, with the corresponding detectors and laser sources.				
**Intravital Microscope**	Intracellular imaging with live organisms	Very high light penetration with multiphoton laser combination	Single application, relatively slow acquisition speeds, very high cost of entry	< 170 nm @ λ_{ex} = 400 nm in H_2O [4]
Intravital microscopes are highly specialised laser scanning confocal microscopes limited to upright configurations attached with an additional multiphoton laser as an excitation source, and often external detectors with increased light sensitivity. Due to these added components over a typical confocal microscope, these configurations are generally very expensive.<br><br>Due to the use of a multiphoton laser as an excitation source, intravital microscopes are able to optically penetrate much deeper into samples (typically in the order of 100's of microns), which is critical when imaging live animals or organisms. The longer wavelengths used by multiphoton lasers allow for both gentle imaging and greater optical penetration over that of shorter wavelength solid-state lasers used in standard confocal microscopes.				
**Super Resolution**	Imaging of sub cellular features, single molecules	Very high resolution, single molecule imaging, some applications have very high acquisition speeds (200+ fps, frames per second)	Very high cost of entry, very large datasets, more in-depth training required, some applications may require special sample preparation	< 50 nm @ λ_{ex} = 400 nm in oil with specialised algorithm and SIM [5]
Super resolution microscopes are another type of highly specialised laser scanning confocal microscope, that are used when imaging extremely small features (typically below 150 nm in resolution). There are several super resolution microscopes available, each with varying resolution improvements and caveats. The simplest in terms of usage is the ZEISS AiryScan, which simply replaces the single point detector with a hexagonal array detector, and with a dedicated algorithm, can improve image resolution by 1.5× with no additional sample preparation. Another technique is STED super resolution, where a "donut" is shone onto the sample along with the excitation laser, helping to quench extraneous stray light emitted from the imaging plane, helping achieve resolutions of up to 100 nm. The drawback of STED, is that two laser beams are simultaneously shone onto the sample, leading to increased photobleaching. Specialised stains can be used to alleviate this issue but require special sample preparation. SIM is another technique, able to achieve a two-fold increase in resolution. SIM microscopes do not require any special sample preparation but have limited penetration depths				

limited typically monolayer cell cultures. SIM microscopes are best suited to observe intra and inter cellular dynamic processes, as they are very speed and resolution. Finally, single molecule super resolution microscopes offer the highest possible resolution of any visible light microscope. Single molecule microscopes require highly specialised sample preparation, where individual molecules are made to "blink" between multiple images. Thousands of these images are then captured and processed and the final output then creates an image allowing users to observe single molecules.				
**Light-sheet**	Whole organism or organ imaging	3D imaging, very large samples, fast acquisition speeds	High cost of entry, very large datasets, samples often need to be cleared	< 100 nm @ λ_{ex} = 400 nm dependent on clearing media [6,7]
Light-sheet microscopes operate differently with respect to sample handling. Samples are typically placed into a chamber and suspended using a post or hook and immersed in H_2O, PBS or clearing media. Samples are often required to be prepared with specialised clearing agents, to ensure the excitation light-sheet is able to penetrate the whole sample and capture the best possible image. This process can be difficult and may also use corrosive agents to achieve the clearing process.<br><br>The ability of a light-sheet microscope to image whole organisms or organs allows for very in-depth analysis of cellular processes.				

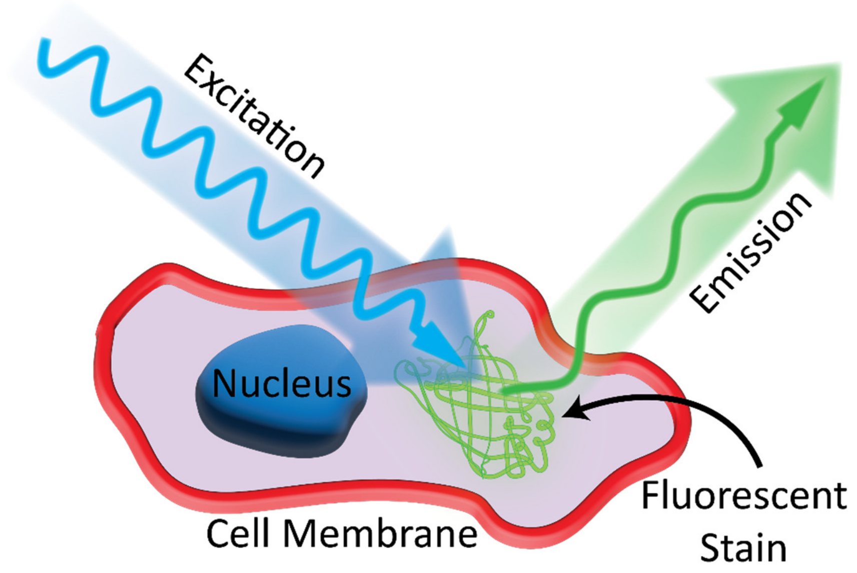

Figure S1: A simplified diagram of fluorescence within a cell. A shorter wavelength excitation light beam is shone onto the cell, where a fluorescence compound is localised. The fluorescence compound then emits a longer wavelength light beam, which can then be imaged.

References

1. Marvin, M. Microscopy apparatus. 19 Dec 1961, 1961.
2. Minsky, M. Memoir on inventing the confocal scanning microscope. *Scanning* **1988**, *10*, 128-138.
3. Pawley, J. *Handbook of biological confocal microscopy*. Springer Science & Business Media: 2006; Vol. 236.
4. Boulch, M.; Grandjean, C.L.; Cazaux, M.; Bousso, P. Tumor immunosurveillance and immunotherapies: A fresh look from intravital imaging. *Trends in immunology* **2019**, *40*, 1022-1034.
5. Kubalová, I.; Němečková, A.; Weisshart, K.; Hřibová, E.; Schubert, V. Comparing super-resolution microscopy techniques to analyze chromosomes. *International Journal of Molecular Sciences* **2021**, *22*, 1903.
6. Held, M.; Santeramo, I.; Wilm, B.; Murray, P.; Lévy, R. Ex vivo live cell tracking in kidney organoids using light sheet fluorescence microscopy. *PLoS One* **2018**, *13*, e0199918.
7. O'Donnell, N.; Dmitriev, R.I. Three-dimensional tissue models and available probes for multi-parametric live cell microscopy: A brief overview. *Multi-Parametric Live Cell Microscopy of 3D Tissue Models* **2017**, 49-67.
